# Isatin–pyridine oxime hybrids as potential reactivators of A-242-inhibited acetylcholinesterase

**DOI:** 10.1007/s00204-026-04449-1

**Published:** 2026-05-22

**Authors:** Amanda M. V. Pinto, Dipanjan Bhattacharyya, Munique C. J. da Silva, Daniel A. S. Kitagawa, Marcelo C. dos Santos, Samir F. de A. Cavalcante, Salim T. Islam, Steven R. LaPlante, Pat Forgione, Joyce S. F. Diz, Tanos C. C. França

**Affiliations:** 1 Laboratory of Molecular Modeling Applied to Chemical and Biological Defense, Military Institute of Engineering, Rio de Janeiro, 22290-270, RJ Brazil; 2 Department of Chemistry and Biochemistry, Concordia University, Montreal, H4B 1R6, QC Canada; 3 Institute of Chemical, Biological, Radiological and Nuclear Defense (IDQBRN), Brazilian Army Technological Center (CTEx), Rio de Janeiro, 23020-470, RJ Brazil; 4 Agency for Technology Management and Innovation (AGITEC), Rio de Janeiro, 23020-470, RJ Brazil; 5 Center for Basic and Applied Research, Faculty of Informatics and Management, University of Hradec Kralove, 50003 Hradec Kralove, Czech Republic; 6 Centre Armand-Frappier Santé Biotechnologie, Institut National de la Recherche Scientifique (INRS), Laval, H7V 1B7, QC Canada; 7 School of Health, Concordia University, Montreal, Quebec, H4B 1R6, QC Canada; 8 Centre for Nanoscience Research (CeNSR), Concordia University, Montreal, H4B 1R6, QC Canada; 9 PROTEO, the Quebec Network for Research on Protein Function, Engineering, and Applications, Université Laval, Quebec, G1V 0A6, QC Canada

**Keywords:** Acetylcholinesterase, Organophosphorus, Surrogates, Inhibitors, A-series, Nerve agents

## Abstract

Three new isatin–pyridine-2-oxime hybrids (AM01–03) were synthesized and experimentally evaluated as reactivators of acetylcholinesterase (AChE) inhibited by a surrogate of the nerve agent A-242, in comparison with their 4-oxime isomers previously reported by our research group. Furthermore, the interactions of these molecules within the AChE/A-242 complex were assessed through docking and molecular dynamics (MD) simulations. Our results show that the 2-oxime isomers, unlike their 4-oxime analogues, can reactivate AChE inhibited by the A-242 surrogate at 100 µM and are even capable of outperforming the commercial reactivator HI-6. These findings suggest that isatin–oxime hybrids may exhibit a specific biological activity—similar to that observed for obidoxime and trimedoxime—in the reactivation of AChE inhibited by A-242.

**Supplementary Information:**

The online version contains supplementary material available at 10.1007/s00204-026-04449-1.

## Introduction

The termination of parasympathetic synaptic transmission depends on acetylcholinesterase (AChE), the enzyme responsible for hydrolysing acetylcholine (ACh) into choline and acetate, thereby regulating neurotransmission. Primarily located in the brain, AChE is also found in erythrocytes and muscle tissue and is evolutionarily conserved across species. Its catalytic activity involves a triad of serine, histidine, and glutamate residues at the active site (Figueroa-Villar et al. 2021; Quinn 1987; Soreq and Seidman 2001). Exposure to organophosphorus compounds (OPs) leads to rapid phosphorylation of the serine residue within this catalytic triad, resulting in irreversible enzyme inactivation. This causes ACh to accumulate, triggering sustained activation of cholinergic pathways (Sussman and Silman 1992; Wang et al. 2022). The overstimulation produces a range of clinical symptoms known as cholinergic crisis and collectively described as SLUDGEM syndrome. Without timely intervention, these effects can escalate to life-threatening complications (Candiotti 2017; Dvir et al. 2010).

Despite their widespread use as pesticides, OPs are highly toxic, posing severe risks to human health and accounting for approximately 300,000 deaths annually worldwide (Chaumont-Olive et al. 2021; Okello et al. 2022; Valiveti et al. 2015). Furthermore, certain OPs are classified as nerve agents, considered the most toxic class of chemical warfare agents (CWAs) (Termeau et al. 2023). Historical incidents—such as the 1995 Tokyo subway attack and the 2017 assassination of Kim Jong-Nam with VX—underscore the ongoing and significant threat posed by these agents. Although the Chemical Weapons Convention (CWC) (https://www.opcw.org/chemical-weapons-convention) aims to limit their proliferation, the use of OPs in armed conflicts and terrorist activities remains a pressing global concern (Brunka et al. 2022; Costanzi et al. 2018; Dvir et al. 2010; Wiener and Hoffman 2004).

**Fig. 1:**
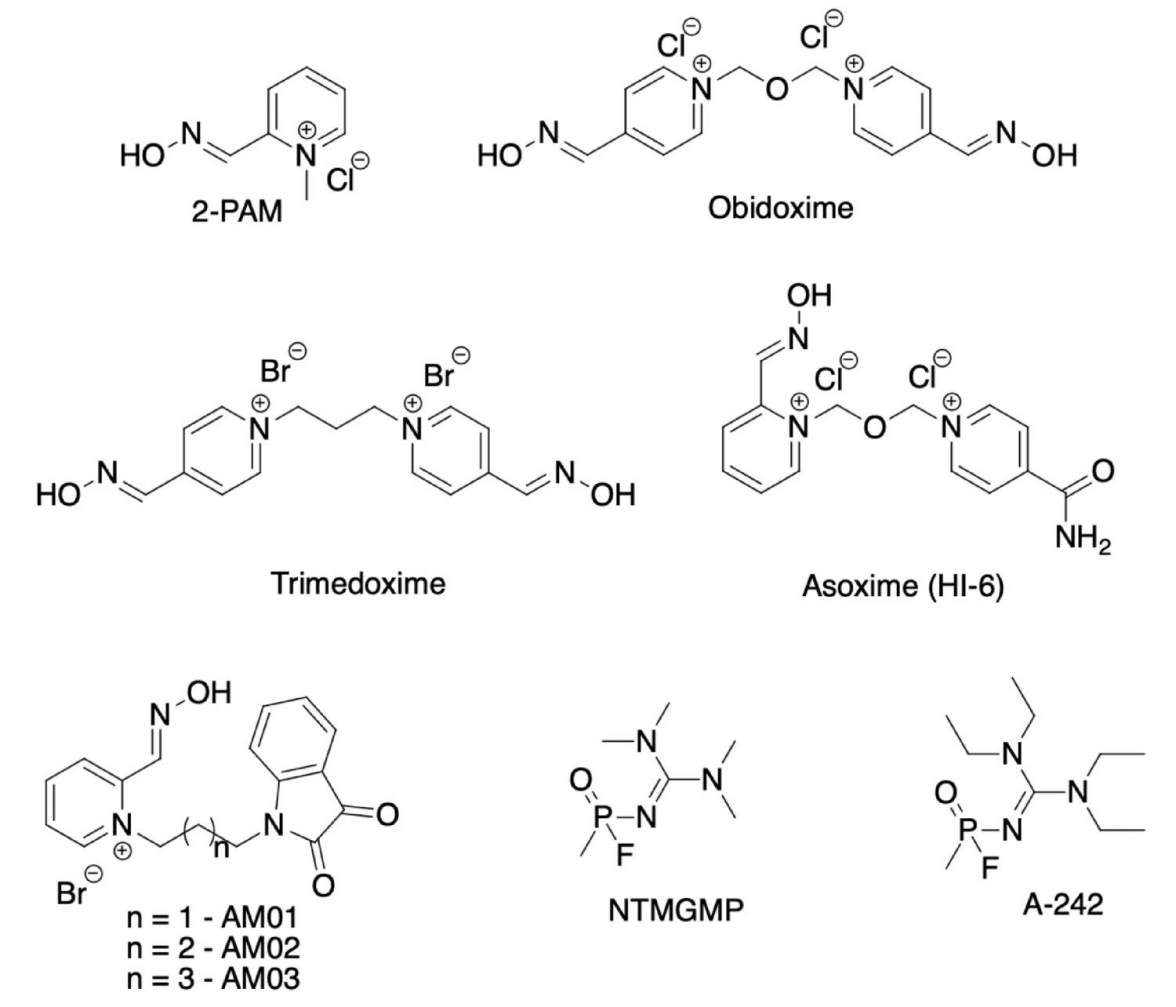
Structures of 2-PAM, obidoxime, trimedoxime, asoxime (HI-6), AM01–03, NTMGMP (Santos et al. 2022a) and A-242

Treatment of OP poisoning typically involves benzodiazepines to manage seizures, atropine to counteract muscarinic effects, and AChE reactivators. However, currently available clinical reactivators—such as pralidoxime (2-PAM), obidoxime, trimedoxime, and asoxime (HI-6) (Fig. 1)—have notable limitations. These include poor permeability across the blood–brain barrier (BBB), inherent toxicity, and a narrow range of efficacy (Cavalcante et al. 2019b; Nepovimova and Kuca 2019). Furthermore, these reactivators must be administered promptly to avoid the “aging” process, which irreversibly inactivates AChE. These challenges highlight the urgent need for novel reactivators with improved pharmacokinetic properties and broader therapeutic effectiveness (Cavalcante et al. 2019b; Cowen et al. 2022; Franca et al. 2019).

Our research group has investigated neutral aryloximes and isatin–oxime derivatives as potential alternative AChE reactivators (Bernardo et al. 2025; Cavalcante et al. 2019b; Kitagawa et al. 2019). Unlike traditional reactivators, these compounds are not biscationic (see Fig. 1). While this structural difference may reduce their intrinsic reactivity, it offers potential advantages, such as better BBB permeability and enhanced interactions within the enzyme’s active site—promising features that could guide the development of more effective reactivators (Kitagawa et al. 2019; Zhao and Tang 2002). Building on this work, we report here the synthesis, docking and molecular dynamics (MD) studies of three new isatin–oxime derivatives (AM01–AM03 in Fig. 1), along with an experimental evaluation of their ability to reactivate AChE inhibited by 4-nitrophenyl *N*-(bis(dimethylamino) methylene)-P-methylphosphonamidate (NTMGMP) a surrogate of the OP A-242 (Fig. 1).

## Materials and methods

### General information

All chemicals were purchased from commercial suppliers and used without further purification. Isatin, *Electrophorus eel* AChE (*Ee*AChE) (C2888, Type V-S, 1000 U/mg protein), pralidoxime iodide, 1,3-dibromopropane, 1,4-dibromobutane, 1,5-dibromopentane, anhydrous potassium carbonate, phosphate salts for buffer preparation, anhydrous sodium sulfate, and dry *N*, *N*-dimethylformamide (DMF) (sealed bottle) were obtained from Sigma-Aldrich Canada (Ontario, Canada) and AKS (Ontario, Canada). NTMGMP was produced *in house* following our previously published protocol (Santos et al. 2022a).

Acetonitrile and methanol for HPLC-DAD-MS analyses were also sourced from Sigma-Aldrich Canada. Deuterated solvents (chloroform-d and DMSO-d₆) were purchased from AKS (Canada). Deuterated chloroform with 0.05% (v/v) TMS was obtained from Cambridge Isotope Laboratories, while chloroform-d without TMS was purchased from Sigma-Aldrich. Purified water was obtained using a Millipore Milli-Q system (18,2 MΩ·cm at 25 °C). Thermal reactions were conducted in a silicon oil bath on a stirrer-hotplate equipped with both a temperature probe and an independent thermometer. Microwave-assisted reactions were carried out in 20 mL microwave vials using a Biotage Initiator™+ microwave synthesizer (400 W magnetron).

Purification was performed by silica gel column chromatography (SiliCycle^®^ F60, 40–63 μm, 60 Å) and monitored by thin-layer chromatography (TLC) using SiliCycle^®^ SiliaPlate^®^ aluminum-backed TLC plates (silica, 200 μm, 20 × 20 cm). GC–MS analyses were conducted on an Agilent 7890 A GC system coupled to an Agilent 5975 C VL MSD with a Triple–Axis Detector. The system employed an HP–588 column coated with (5%–phenyl)–methylpolysiloxane, using helium as the carrier gas. Samples were dissolved in either dichloromethane (DCM) or methanol (MeOH).

^1^H NMR spectra were recorded on a Varian VNMRS-500 (500 MHz) and a Bruker-300 (300 MHz), while ^13^C NMR spectra were acquired on a Varian VNMRS-500 with a 125 MHz probe.

96-well microplates were purchased from Kasvi Brazil (São José dos Pinhais, Paraná, Brazil). Gilson single-channel pipettes were obtained from Gilson Inc, (Middleton, WI, USA), and Eppendorf 8-channel pipettes from Eppendorf Brazil (São Paulo, Brazil). Ellman’s assays were performed in quadruplicate, across two independent experiments, and by at least three operators, with measurements recorded at 25 ± 2°C. All calculations were performed using Microsoft Excel 2010^®^.

Glassware and disposable materials exposed to OP compounds were decontaminated in a 10% (w/v) NaOH and 10% (w/v) NaClO aqueous solution for 48 h at room temperature in a fume hood prior to proper disposal.

pKa and logP estimations were performed using the ChemAxon Online Suite (Swain 2012), while drug-likeness scores (DS) were calculated using OSIRIS Property Explorer (https://www.organic-chemistry.org/prog/peo/). GC data purity assessments were carried out using Agilent ChemStation software (E,02,02,1431) via area integration,

### Ellman’s assay

The method employed was a modified version of the protocol described before by our research group (Cavalcanteet al. 2018; Santos et al. 2022a), adapted for use with 96-well microplates. Initially, 10 µL of inhibitor solution (20 µM of NTMGMP) or 10 µL of 0.12 M phosphate-buffered saline (PBS, pH 7.6 ± 0.01) for negative controls was added to each well, followed by 70 µL of AChE solution (2.14 U/mL per well) and 80 µL of DTNB (1 mM). The mixture was incubated for 10 min to allow enzyme inhibition to occur. Subsequently, 20 µL of the test compounds at final concentrations of 100, 10, and 1 µM, or 20 µL of PBS for control wells (A₀ for the uninhibited enzyme and A_i_ for the inhibited enzyme without reactivator), was added to initiate the reactivation process. This reaction was allowed to proceed for 30 min. Finally, 20 µL of ATCI (1 mM) was added, and absorbance (A_r_) was measured at 412 nm at various time intervals. The percentage of AChE inhibition and reactivation was calculated using Eqs. (1) and (2). None of the synthesized compounds showed absorbance at 412 nm or reacted with assay reagents, confirming the absence of oximolysis under the tested conditions.

1 $$\:{\%}\mathrm{I}=100\times \:\left(\frac{{\mathrm{A}}_{0}-{\mathrm{A}}_{\mathrm{i}}}{{\mathrm{A}}_{0}}\right)$$

2 $$\:{\%}\mathrm{R}=\left[1-\left(\frac{{\mathrm{A}}_{0}-{\mathrm{A}}_{\mathrm{r}}}{{\mathrm{A}}_{0}-{\mathrm{A}}_{\mathrm{i}}}\right)\right] \times \:100$$

### Synthesis of the isatin-oxime derivatives

**Scheme 1:**
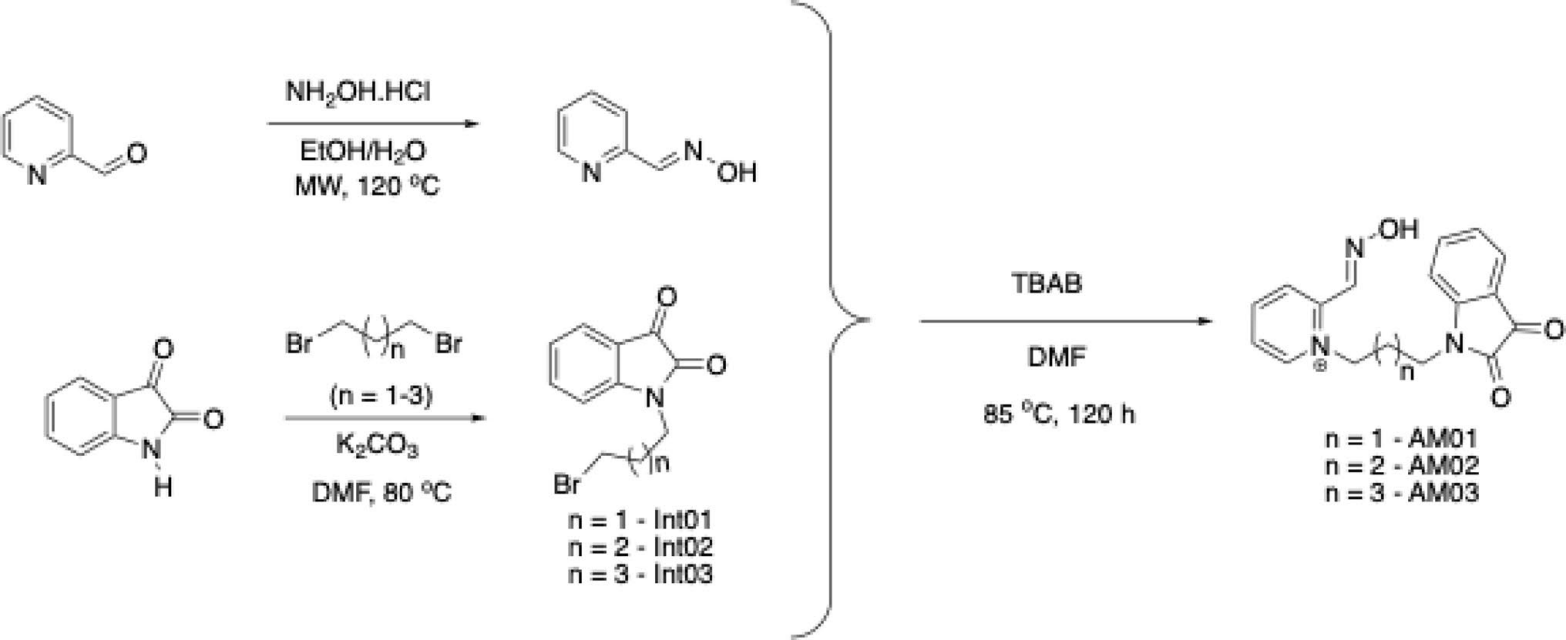
Synthesis of AM01–AM03

The synthetic route was designed as a convergent, three-step process, as illustrated in Scheme 1. The procedure for obtaining the neutral oxime intermediates (Int01–Int03) was adapted from methods described before by our research group (Cavalcante et al. 2019a; Kitagawa et al. 2021). In the first step, a microwave reaction vial equipped with a magnetic stir bar was charged with 10 mmol of 2-pyridine carbaldehyde, 20 mmol of hydroxylamine hydrochloride, and 15 mL of a 50% v/v water/ethanol solution. The vial was sealed and stirred at room temperature for 60 s, followed by microwave irradiation at 120 °C under automatic power control for 1 hour. Upon completion, the reaction mixture was concentrated under reduced pressure using a rotary evaporator at 50 °C until no further solvent remained. The resulting residue was extracted with ethyl acetate (10 mL) and water (20 mL). The organic layer was collected, dried over anhydrous sodium sulfate, filtered, and concentrated under reduced pressure to afford the oxime intermediates as solids of high purity, as confirmed by TLC and GC analysis. The second step involved the synthesis of the isatin derivatives bearing alkyl linkers. Under an inert atmosphere, a sealed tube was charged with 1 mmol of isatin, 3.5 mmol of potassium carbonate, 10 mmol of a 1,ω-dihaloalkane, and DMF at a concentration of 0.5 mol/L relative to the limiting reagent. The mixture was stirred at 80 °C for 6 h. Reaction progress was monitored by silica gel TLC using a 30% ethyl acetate/hexanes eluent. Upon completion, the crude product was purified by column chromatography with 35% ethyl acetate/hexanes as the eluent. In the final step, a sealed tube under an inert atmosphere was charged with 0.2 mmol of the oxime, 0.4 mmol of 2-(hydroxyiminomethyl)pyridine, 3 mL of DMF, and 0.05 equivalents of tetra-*n*-butylammonium bromide (TBAB). The reaction mixture was stirred at 85 °C for 120 h. Progress was monitored every 12 h by silica gel TLC using 80% ethyl acetate/hexanes as the mobile phase. After completion, the reaction mixture was evaporated under reduced pressure, and the crude product was purified by preparative TLC using 80% ethyl acetate/hexanes as the eluent. Characterization data, including ^1^H and ^13^C NMR spectra, as well as LC–MS profiles, are provided in the Supplementary Material (Figs. S1–S9).

### Docking studies

As before (Santos et al. 2022a, b), the model of human AChE (*Hss*AChE) complexed to A-242 used for the modeling studies was constructed based on the crystallographic structure of recombinant *Hss*AChE inhibited by VX, available in the RCSB-Protein Data Bank (Berman et al. 2002) with the code 6CQW (Bester et al. 2018). The model was imported into the Molecular Operating Environment (MOE^®^) platform and prepared with the *QuickPrep* routine, which refined bond distances, corrected geometric parameters, and assigned protonation states consistent with physiological pH.

The 3D structures of HI-6 and the oximes AM01–AM03 (Fig. 1) were constructed with the MOE^®^ *Builder *module, stored in a database (.mdb format), and subsequently optimized to ensure accurate geometries, charges, and protonation profiles. This compound library was then docked into copies of the AChE/A-242 complex using MOE^®^’s *Dock* tool. The docking space was centred on the HI-6 binding pocket of each receptor, with ligand placement performed by the Triangle Matcher algorithm under rigid receptor conditions.

For each ligand, the 100 best poses ranked by the London dG scoring function were retained, followed by rescoring with the GBVI/WSA dG method. From these, the 10 top-scoring conformations were selected for further analysis. Docking protocol validation was performed via re-docking of HI-6 into its original binding site in the crystallographic structure, yielding results consistent with previous studies (Santos et al. 2022a, b; Vieira et al. 2023). Finally, the Near Attack Conformation (NAC) approach (França et al. 2023) was applied to identify the most promising ligand conformations for further investigation.

### Molecular dynamics simulations

As previously reported (Franca et al. 2025; França et al. 2024, 2025; Neto et al. 2025), MD simulations were performed in triplicate using the *Dynamics* tool within the MOE^®^ software package. The input structures were the AChE/A-242/Oxime complexes derived from the best-ranked docking poses and were further prepared for MD using parameters compatible with the NAMD software (Nelson et al. 1996) and the AMBER10:EHT force field (Case et al. 2008). A 10 Å cutoff was applied for electrostatic interactions, and a range of 8–10 kcal·mol⁻¹ was used for van der Waals (VdW) interactions. Each complex was centred within a cubic simulation box containing approximately 30,000 water molecules and neutralized with the appropriate counterions. The system preparation included 10 ps of energy minimization, followed by 100 ps of simulation under NPT conditions, and additional 200 ps under NVT conditions. The production phase consisted of 200 ns of unconstrained MD simulation. Binding energies were calculated using the Dock Score function based on MOE^®^’s *in-house* force field, specifically employing the GBVI/WSA ΔG scoring method (Labute 2010). MD trajectories were analyzed using the *md_analysis* tool and *Database Viewer (DBV)* menu in MOE^®^, as well as the open-source Visual Molecular Dynamics (VMD) software (Humphrey et al. 1996). Plots of RMSD, RMSF, R_θ_, and binding energy were generated using GraphPad Prism 7 (GraphPad Software^®^, San Diego, CA, USA).

**Fig. 2:**
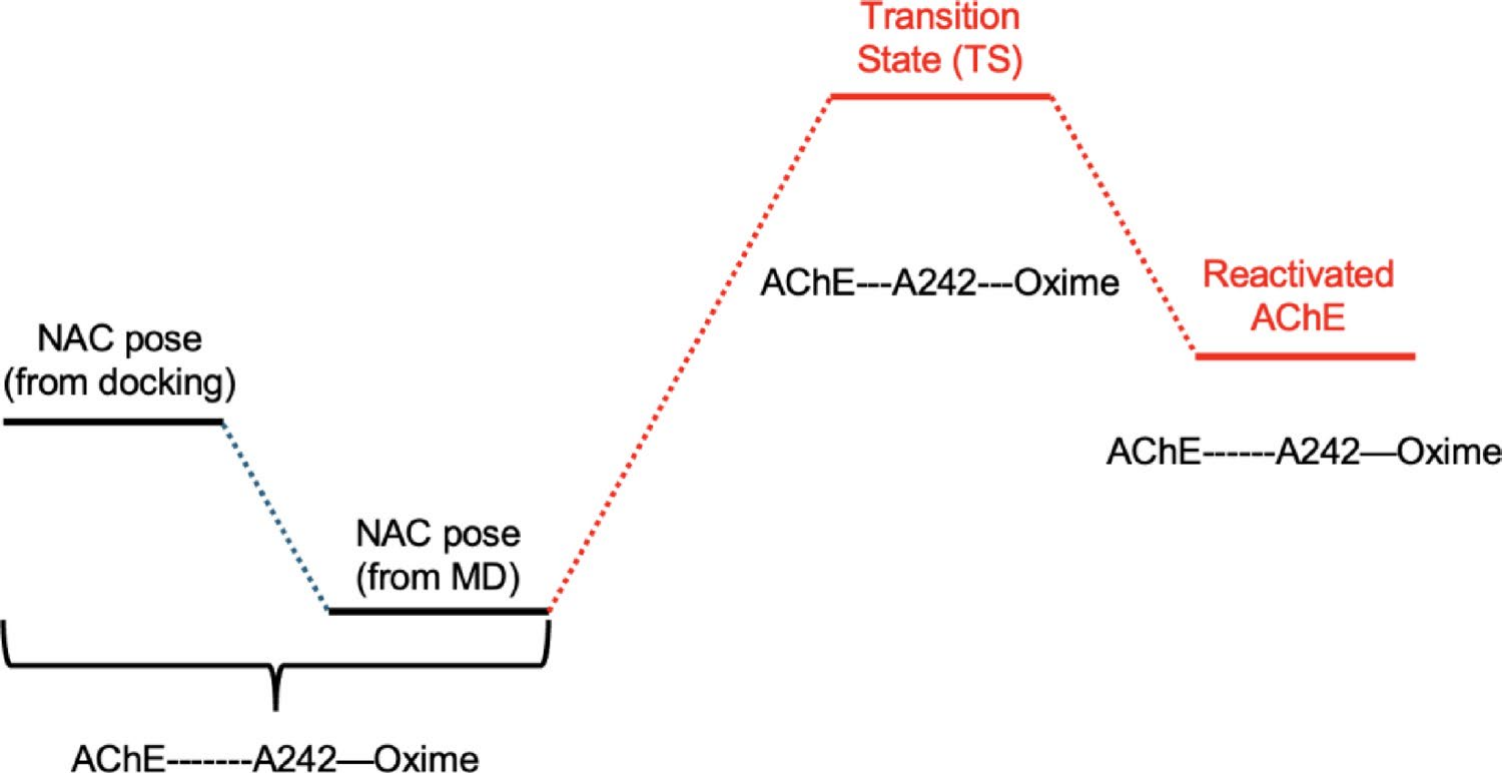
Qualitative representation of the theoretical approach used in this work

A qualitative representation of the reaction coordinate and the strategy employed in this work to identify a refined near-attack conformation (NAC) is presented in Fig. 2. The selected NAC will serve as the starting structure for future, more detailed theoretical investigations using quantum mechanics/molecular mechanics (QM/MM) methods, which correspond to the red-highlighted region of the diagram. These advanced simulations are essential for elucidating the still not fully understood mechanism of acetylcholinesterase (AChE) reactivation by oximes.

## Results and discussion

### Synthesis

The synthesis of *N*-(ω-haloalkyl)isatins Int01–03 was achieved with good yields (see Table 1) and purity, although difficulties were encountered in removing excess linker, particularly 1,5-dibromohexane (Int03), as noted in the literature (Kitagawa et al. 2021). While chromatographic techniques are often cited as effective for removing unreacted linkers, Kitagawa and co-workers (Kitagawa et al. 2021) reported that variations in linker quantity, the use of nucleophilic catalysts, and temperature reduction failed to overcome this issue. Furthermore, decreasing the linker concentration resulted in the formation of by-products. These factors likely contributed to the lower yield of the final product AM03. In contrast, AM01 and AM02, which incorporate shorter linkers, were obtained in higher yields (Table 1).

**Table 1:** Summary of the synthesis with reaction time and yield

Linker	Int01–03		AM01–03	
	Reaction time (h)	Yield (%)	Reaction time (h)	Yield (%)
–(CH_2_)_3−_	4	49	120	67
–(CH_2_)_4−_	6	45	120	65
–(CH_2_)_5−_	6	41	125	33

### In vitro assays

Compounds AM01–AM03 were evaluated for their potential to reactivate AChE inhibited by NTMGMP, using Ellman’s spectrophotometric method. At a concentration of 100 µM, all three compounds exhibited reactivation, with AM01 showing the highest efficacy (13.7 ± 0.2%), followed by AM02 (11.2 ± 0.1%) and AM03 (9.7 ± 0.1%) (Table 2). According to the threshold proposed by Oh and co-workers (Oh et al. 2006), rates above 5% are considered indicative of meaningful AChE reactivation. Notably, AM01–03 surpassed this threshold at 100 µM.

For comparative purposes, the AM01–03 analogues with the oxime group at position 4, SC412, DK61, and DK67, previously described by our group (da Silva et al. 2025; Kitagawa et al. 2019, 2021), were tested under identical conditions, alongside the clinically used oxime HI-6. Interestingly, the reactivation profiles of AM01–AM03 at 100 µM were markedly superior to those of SC412, DK61, and DK67, which displayed reactivation rates between 0.6 and 4.4% at the same concentration. HI-6 showed moderate activity (6.0 ± 3%) at 100 µM, consistent with literature values (Santos et al. 2022a).

A structure–activity relationship (SAR) analysis revealed that the position of the oxime group on the pyridine ring critically influences reactivation potency. Specifically, relocating the oxime from position 4 to position 2 resulted in a marked increase in reactivation efficacy. This trend was particularly evident at 100 µM, where AM01–03 outperformed its 4-oxime counterparts. These findings suggest a biological preference for the 2-oxime configuration in the context of NTMGMP-inhibited AChE, reminiscent of the behaviour observed for bispyridinium oximes such as obidoxime and trimedoxime, yet contrasting with the monopyridinium oxime 2-PAM (Santos et al. 2022a). Interestingly, for paraoxon and the VX-surrogate NEMP (data not shown), we observed the opposite results, supporting the current knowledge that there is no universal antidote so far.

**Table 2:** Reactivation of AChE inhibited by NTMGMP expressed as % (mean ± standard deviation)

	1 µM	10 µM	100 µM
AM01	2.2 ± 0.1	2.5 ± 0.1	13.7 ± 0.2
AM02	0.8 ± 0.1	0.0 ± 0.1	11.2 ± 0.1
AM03	0.7 ± 0.1	2.5 ± 0.1	9.7 ± 0.1
SC412	1.3 ± 0.0	3.7 ± 0.1	4.4 ± 0.1
DK67	1.9 ± 0.1	3.2 ± 0.1	0.6 ± 0.1
DK61	2.4 ± 0.1	2.1 ± 0.1	2.9 ± 0.1
HI-6	0.0 ± 0.0	0.0 ± 0.0	6.0 ± 3.0

### Docking

Molecular docking studies were performed to gain structural insights into the potential reactivation mechanism of compounds AM01–03 within AChE active site gorge, using the enzyme covalently inhibited by A-242 (AChE/A242). As summarized in Table 3, all three compounds exhibited binding energy values comparable to that of the reference oxime HI-6, suggesting favourable complementarity with the inhibited enzyme.

**Fig. 3:**
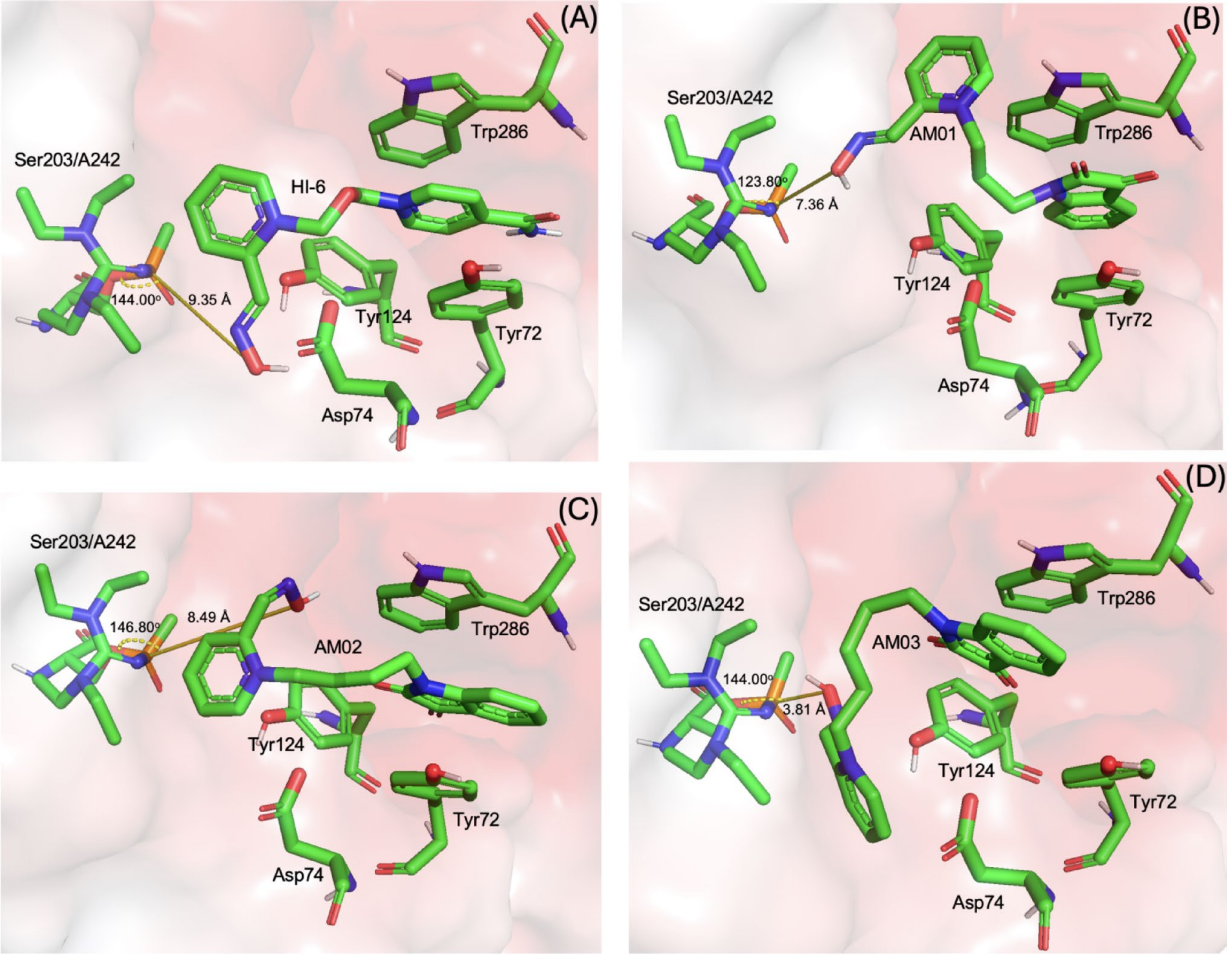
Best poses for each oxime inside the AChE/A-242 complex, **A** HI-6, **B** AM01, **C** AM02, **D** AM03

Analysis of the top-scoring poses revealed that the isatin moiety of AM01–03 consistently engaged in π-stacking interactions with the peripheral anionic site residues Tyr72 and Trp286 (Fig. 3), a binding mode reminiscent of that observed for HI-6. These interactions may contribute to stabilizing the ligand within the gorge and orienting the oxime group for nucleophilic attack on the Ser203–OP conjugate.

To further assess the conformational suitability of these compounds for reactivation, we calculated the distance from the oxime oxygen to the phosphorus atom of the A-242 adduct (d_OP_) and the attack angle (θ_OPO_), defined as the O_oxime_–P_OP_–O_Ser203_ angle. The ratio d_OP_/θ_OPO_, R_θ_, was used as a descriptor to estimate the geometrical predisposition for nucleophilic attack, with values closer to 54.55 indicating a more favourable orientation (França et al. 2023).

When compared to HI-6, AM01–03 showed R_θ_ values closer to 54.55 (Table 3), indicating a better alignment for nucleophilic attack. Particularly the conserved π-stacking interactions, suggest that AM01–03 occupy a similar region within the active site gorge and may adopt conformations conducive to reactivation consistent with the NAC hypothesis (França et al. 2023).

These docking data, combined with the experimental reactivation profiles (Table 2), support the notion that AM01–03 possess structural features that warrant further optimization, particularly in fine-tuning the oxime positioning to achieve an orientation more closely resembling that of HI-6.

**Table 3:** Molecular docking results

Ligand	Energy (Kcal/mol)	d_OP_ (Å)	θ_OPO_	*R*_θ_ (Å)
HI-6	−7.54	9.35	130.40	13.95
AM01	−7.23	7.36	123.80	16.82
AM02	−7.61	8.49	146.80	17.29
AM03	−7.17	3.81	144.00	37.80

### Molecular dynamics simulations

In MD simulations, the selection of representative conformations is a critical step for achieving both methodological soundness and computational practicality in downstream analyses. Because a 200-ns simulation generates a substantial number of frames, evaluating the entire trajectory can be unnecessarily repetitive and computationally demanding. To overcome this limitation, 800 frames were evenly sampled throughout the trajectory, ensuring an unbiased yet tractable representation of the conformational landscape explored.

Beyond uniform sampling, a range of advanced statistical approaches—including statistical inefficiency analysis (Jemai et al. 2026a, b; Ramalho et al. 2010a, b), Principal Component Analysis (PCA) (Jemai et al. 2026b), and wavelet-based methods (Goncalves et al. 2022)—have been introduced to identify frames of greater structural relevance. These strategies reduce the complexity of trajectory data, highlight major conformational changes, and enable more targeted interpretation using a smaller subset of structures. Such approaches are especially advantageous in extended simulations, where a significant proportion of frames may contribute limited additional information.

**Fig. 4:**
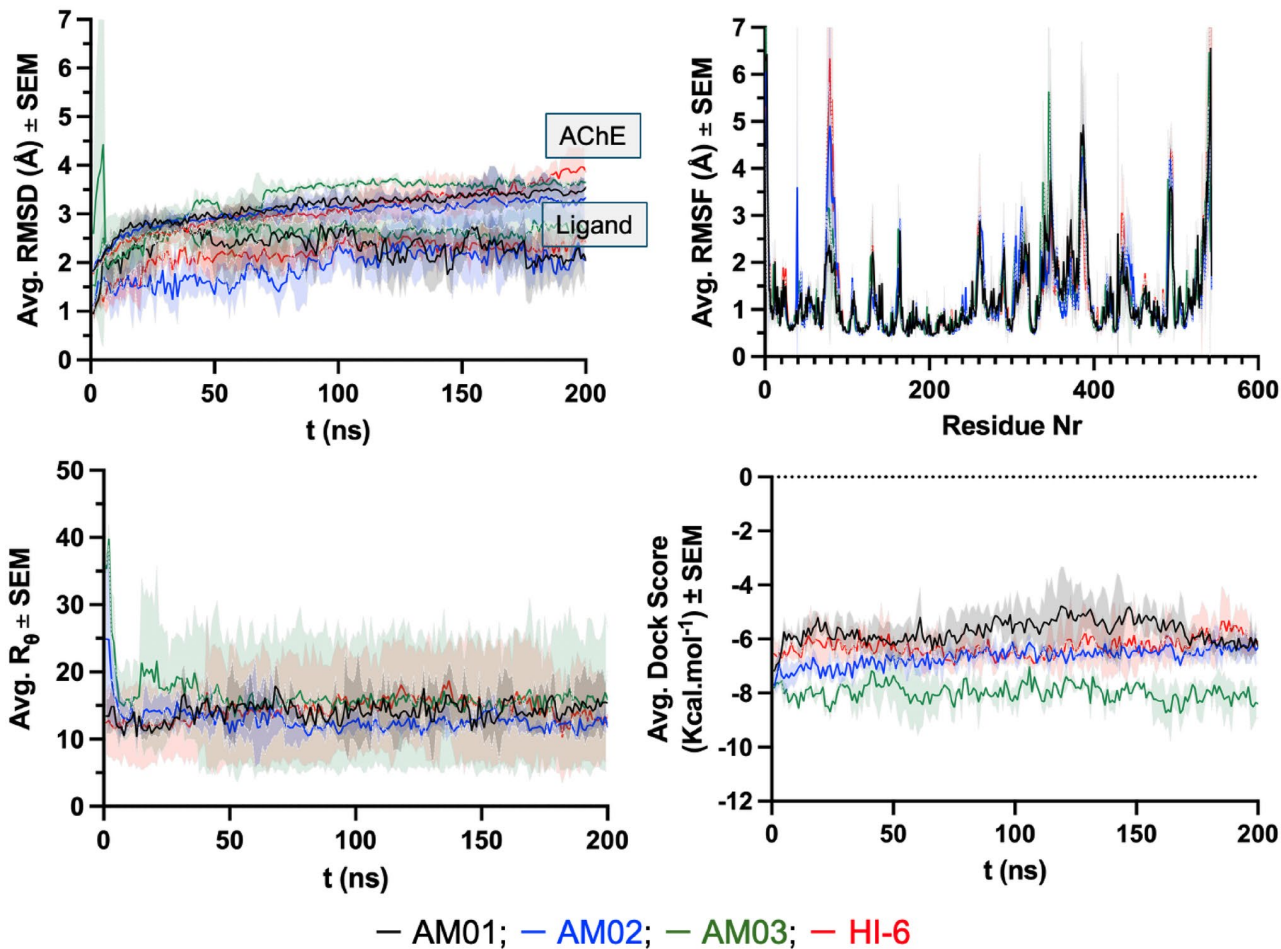
MD results for the HssAChE/A-242/Oxime complexes. Each line represents the mean value between the three MD replicates, and the lighter colour shadows above and below the line represents the standard error of the mean (SEM)

The results of MD simulations are illustrated in the plots of RMSD, RMSF, R_θ_, and binding energy for the HssAChE/A-242/Oxime complexes (Fig. 4). The RMSD profiles indicate that all systems reached stabilization early in the simulations, with no fluctuations exceeding 4.0 Å for the protein or 3.0 Å for the ligands. Local fluctuations throughout the simulations were almost negligible for the protein and below 1.0 Å for ligands. The RMSF plots demonstrated similar fluctuation profiles across all systems, with deviations in α-helices and β-sheet regions remaining below 2.0 Å. This supports the overall stability of the simulations and indicates that ligand binding does not significantly disrupt protein conformation.

The R_θ_ descriptor, which integrates distance and attack angle, provides a more complete assessment of conformational readiness for nucleophilic attack. The averaged R_θ_ values for HI-6, AM01 and AM02 (14.37, 14.10 and 12.86, respectively) didn’t deviate much from the initial values observed in the docking studies (see Table 3). AM03, on the other hand, showed a more significant variation with an averaged R_θ_ = 16.33. This suggests the stabilization of this oxime on a conformation less favourable to the NAC and reflects the worse experimental result when compared to AM01 and AM02 (See Table 2).

Finally, the binding energy plots showed consistently negative values throughout the simulations. AM02 and AM03 showed the lowest average values of − 6.67 and − 7.98 kcal mol^− 1^, respectively, followed by HI-6 (− 6.28 kcal mol^− 1^) and AM01 (− 5.71 kcal mol^− 1^). These results qualitatively support the findings from both docking and MD simulations, indicating that all ligands can effectively bind to and remain stable within the AChE active site.

Integration of all descriptors reveals distinct conformational sampling patterns, AM01 and AM02 maintains more stable profiles across all metrics: RMSD, R_θ_, and binding energy. This internal consistency indicates a better-defined, persistent binding mode with the oxime group consistently oriented for attack. AM03 displays the most heterogeneous profile. It’s excellent binding affinity is offset by poorer geometrical descriptors, that might explain the lower reactivation efficacy, it binds tightly but in a less-productive orientation.

The better performance of AM01 and AM02 can be attributed to persistent π-stacking interactions between the isatin moiety and the peripheral anionic site residues Tyr72 and Trp286 (see Fig. 2), which anchor the ligand in a productive orientation. AM03, despite its strong binding affinity, appears to be unable to maintain the isatin moiety between Tyr72 and Trp286 due the longer linker (five atoms) and adopts a binding mode where the oxime group points away from the phosphorus or approaches at a suboptimal angle, likely resulting from the different positioning of the oxime group, which alters the vectorial alignment required for nucleophilic attack.

Mechanistically, when interpreting the differences relative to the 4-oxime isomers previously reported by our research group (da Silva et al. 2025; Kitagawa et al. 2019, 2021), one may infer that the electronic effects resulting from shifting the oxime group position introduce significant changes in pKa and, consequently, affect nucleophilicity. At the 4-position, the oxime is para-like relative to the ring nitrogen, allowing stronger and more symmetric resonance interactions. In contrast, at the 2-position, the oxime is ortho-like, which alters the inductive/resonance balance and often increases local electronic perturbation.

Additionally, at the 2-position, the oxime hydroxyl group can often form an intramolecular hydrogen bond with the pyridine nitrogen, thereby stabilizing a specific tautomeric or protonation state. This interaction is not possible, or is considerably weaker, at the 4-position. As a result, the acidity of the oxime OH, the basicity of the pyridine nitrogen, and the overall microscopic pKa values can be significantly altered.

Finally, from a steric perspective, the 2-substituted analogue may adopt a different spatial orientation, which can affect conjugation and electron delocalization. Reduced conjugation often leads to measurable changes in acidity/basicity relationships.

## Conclusion

The synthesis and characterization of three new isatin–pyridine-2-oxime hybrids (AM01–AM03) were successfully accomplished. Reactivation assays against AChE inhibited by the A-242 surrogate NTMGMP (Santos et al. 2022a) revealed that the three compounds exhibited meaningful reactivation at a concentration of 100 µM while their pyridine-4-oxime analogues failed to reactivate the AChE/NTMGMP complex. This result points to a preference for the 2-oxime configuration in the context of NTMGMP-inhibited AChE.

The docking studies revealed that AM01–AM03 can adopt poses within the AChE active site comparable to the reference oxime HI-6, with the isatin moiety engaging in π-stacking interactions with Tyr72 and Trp286. However, MD simulations results suggest that AM03 seems to adopt a distinct binding mode which alters the vectorial alignment required for nucleophilic attack. This might explain the worse results observed for AM03 compared to AM01 and AM02 and points to a preferential linker size of 3 or 4 atoms for the reactivation of the AChE/A-242 complex by these compounds.

Collectively, these findings support the continued exploration of isatin–pyridine oxime hybrids as AChE reactivators and suggest that further optimization should focus on rigidifying the linker to pre-organize the oxime group in an orientation that drives R_θ_ toward the theoretical optimum. The 2-oxime configuration appears intrinsically superior to the 4-oxime configuration for AChE reactivation, reinforcing the SAR observed for bispyridinium oximes.

## Supplementary Information

Below is the link to the electronic supplementary material.

Supplementary Material 1

## Declarations

### One sentence summary

Isatin–Pyridine Oxime Hybrids qualify as potential reactivators for AChE inhibition by A-242.
