# Supplementary material for: Isatin–pyridine oxime hybrids as potential reactivators of A-242-inhibited acetylcholinesterase
Source: Arch Toxicol. 2026 May 22;100(9):4077–87. doi: 10.1007/s00204-026-04449-1 (PMC13453993; doi:10.1007/s00204-026-04449-1)
Supplement: Supplementary file 1 [file 204_2026_4449_MOESM1_ESM.docx]

**Supplementary Material**

**Isatin–Pyridine Oxime Hybrids as Potential Reactivators of A-242–Inhibited Acetylcholinesterase**

Amanda M. V. Pinto^1,2^, Dipanjan Bhattacharyya^2,3,4^, Munique C. J. da Silva^1,5^, Daniel A. S. Kitagawa^6^, Marcelo C. dos Santos^1^, Samir F, de A, Cavalcante^5,7,*^, Salim T. Islam,^8,9^ Steven R. LaPlante^8,9^, Pat Forgione^2,3,4^, Joyce S. F. Diz^1,5^, Tanos C. C. França^1,5,8,9,*^

^1^Laboratory of Molecular Modeling Applied to Chemical and Biological Defense, Military Institute of Engineering; Rio de Janeiro, 22290-270, RJ, Brazil.

^2^Department of Chemistry and Biochemistry, Concordia University, Montreal, H4B 1R6, QC, Canada.

^3^School of Health, Concordia University, Montreal, H4B 1R6, QC, Canada.

^4^Centre for Nanoscience Research (CeNSR), Concordia University, Montreal, H4B 1R6, QC, Canada.

^5^Institute of Chemical, Biological, Radiological and Nuclear Defense (IDQBRN), Brazilian Army Technological Center (CTEx); Rio de Janeiro, 23020-470, RJ, Brazil.

^6^Agency for Technology Management and Innovation (AGITEC), Rio de Janeiro, 23020-470, RJ, Brazil.

^7^Center for Basic and Applied Research, Faculty of Informatics and Management, University of Hradec Kralove, 50003 Hradec Kralove, Czech Republic.

^8^Institut National de la Recherche Scientifique (INRS), Centre Armand-Frappier Santé Biotechnologie, Pasteur Network, Laval, QC H7V 1B7, Canada.

^9^PROTEO, the Quebec Network for Research on Protein Function, Engineering, and Applications, Universite Laval, Quebec, QC G1V 0A6, Canada.

**Characterization data for compounds AM01-AM03.**

(E)-1-(3-(2,3-dioxoindolin-1-yl)propyl)-2-((hydroxyimino)methyl)pyridin-1-ium (AM01)

Yield: 68%; ^13^C NMR (126 MHz, CDCl_3_): δ 183.33, 161.03, 158.25, 151.68, 150.71, 150.58, 149.38, 138.42, 136.87, 124.11, 123.84, 117.63, 110.02, 63.04,39.71, 25.96, 23.89. ^1^H NMR (500 MHz, cdcl_3_): δ 9.45 (s, 2H), 8.64 (d, *J* = 4.9 Hz, 2H), 8.31 (s, 1H), 8.07 (d, *J* = 12.8 Hz, 1H), 7.84 – 7.79 (m, 2H), 7.65 – 7.56 (m, 2H), 4.25 (dt, *J* = 20.7, 5.9 Hz, 3H), 3.86 (t, *J* = 7.1 Hz, 1H), 2.15 – 2.03 (m, 1H), 1.86 – 1.73 (m, 3H). HPLC-MS-ESI: 310.1 (M^+^).

**Figure S1**. ^1^H NMR spectra of AM01.

**Figure S2**. ^13^C NMR spectra of AM01.

**Figure S3**. MS spectra of AM01.

(E)-1-(4-(2,3-dioxoindolin-1-yl)butyl)-2-((hydroxyimino)methyl)pyridin-1-ium (AM02)

Yield: 68%; ^13^C NMR (126 MHz, CDCl_3_) δ 183.33, 161.01, 158.24, 151.73, 150.71, 150.42, 149.43, 138.40, 136.78, 124.09, 123.83, 117.62, 110.01, 63.03, 39.71, 31.59, 29.05, 25.96. ^1^H NMR (500 MHz, CDCl_3_) δ 9.37 (s, 2H), 8.64 (ddd, *J* = 5.0, 1.8, 1.0 Hz, 2H), 8.32 (s, 2H), 8.06 (d, *J* = 0.9 Hz, 1H), 7.82 (dt, *J* = 8.0, 1.1 Hz, 2H), 7.73 (td, *J* = 7.7, 1.8 Hz, 2H), 7.65 – 7.55 (m, 2H), 7.30 (ddd, *J* = 7.5, 4.9, 1.2 Hz, 2H), 7.13 (td, *J* = 7.5, 0.8 Hz, 1H), 6.91 (dt, *J* = 7.8, 0.8 Hz, 1H), 4.23 (td, *J* = 6.1, 0.9 Hz, 2H), 3.84 – 3.71 (m, 2H). HPLC-MS-ESI: 368.4 (M^+^).

**Figure S4**. ^1^H NMR spectra of AM02.

**Figure S5**. ^13^C NMR spectra of AM02.

**Figure S6**. LC-MS spectra of AM02.

(E)-1-(5-(2,3-dioxoindolin-1-yl)pentyl)-2-((hydroxyimino)methyl)pyridin-1-ium (AM03)

Yield: 76%; ^13^C NMR (126 MHz, CDCl_3_) δ 183.47, 161.12, 158.20, 151.70, 150.85, 150.53, 149.48, 138.36, 136.75, 125.58, 124.10, 123.75, 121.00, 117.61, 110.05, 63.49, 39.97, 28.06, 26.84. ^1^H NMR (500 MHz, cdcl_3_) δ 9.11 (s, 2H), 8.66 – 8.61 (m, 2H), 8.31 (s, 2H), 8.05 (s, 1H), 7.82 (d, *J* = 7.9 Hz, 2H), 7.73 (td, *J* = 7.7, 1.7 Hz, 2H), 7.65 – 7.56 (m, 2H), 7.33 – 7.26 (m, 2H), 7.13 (t, *J* = 7.5 Hz, 1H), 6.90 (d, *J* = 7.9 Hz, 1H), 4.18 (t, *J* = 6.5 Hz, 2H), 3.75 (t, *J* = 7.3 Hz, 2H), 1.75 – 1.61 (m, 1H). HPLC-MS-ESI: 338 (M^+^).

**Figure S7**. ^1^H NMR spectra of AM03.

**Figure S8**. ^13^C NMR spectra of AM03.

**Figure S9**. LC-MS spectra of AM03.
